# The Elusive Legitimacy of EU Soft Law: An Analysis of Consultation and Participation in the Process of Adopting COVID-19 Soft Law in the EU

**DOI:** 10.1017/err.2020.119

**Published:** 2021-03

**Authors:** Mariolina ELIANTONIO, Oana ŞTEFAN

**Affiliations:** * University of Maastricht, The Netherlands; email: m.eliantonio@maastrichtuniversity.nl.; ** King’s College London, UK.

## Abstract

This article takes issue with the legitimacy of EU soft law instruments issued to deal with the COVID-19 crisis. Up to August 2020, we identified a total of 197 such instruments, and analysed the procedures for their adoption. We found little evidence of parliamentary involvement or stakeholder consultation, with COVID-19 soft law replicating decision-making patterns which have been constantly criticised in the literature as illegitimate and opaque. Giving due consideration to the exceptional nature of these measures, the article suggests some quick fixes which might increase, ex post factum, the legitimacy of these instruments.

## I. Introduction

Soft law was the immediate go-to in order to deal with the COVID-19 crisis at the EU level. Soft law instruments are generally defined as rules of conduct that have no legal force but may have legal and practical effects;^1^ they have been employed since March 2020 to set out emergency arrangements for most European Union (EU) policies. In sectors such as competition and state aid, the consequences of the health crisis were dealt with exclusively through soft law.^2^ A Temporary Framework^3^ set out the conditions under which an impressive body of national measures, valued at around €3 trillion at the time of writing,^4^ have been approved by the Commission since the beginning of the pandemic. In general, EU soft law has been adopted for the management of the pandemic for issues as diverse as dealing with seasonal workers in the EU in the context of the COVID-19 outbreak,^5^ to providing guidance on how to restore transport^6^ and tourism^7^ services, and, most recently, on a common approach to restrictions of free movement.^8^

The recourse to soft law in a time of crisis is understandable, given its flexibility and speedy adoption processes.^9^ Yet as experiences from former EU crises show, slipping into informality comes with important price tags in terms of the rule of law, as discussed by Kilpatrick in relation to the financial crisis.^10^ The dubious democratic credentials of soft law are well known. Writing about the soft ways in which the financial crisis was regulated, Dawson and de Witte noted that this altered the constitutional balance in Europe, warning that “especially at a time when the Union is venturing into redistributive politics, it needs to carefully protect its input legitimacy”.^11^ Kjaer agrees, while noting that a crisis of the law is undesirable as it might translate into a return to Europe’s grim past, comparing the post-financial crisis with the inter-war period.^12^ Shocks such as the financial crisis or the current COVID-19 crisis are thus risky for European integration, with Dawson and de Witte concurring with Habermas that another crisis could result in either a slippage into executive federalism or a turn to a fully fledged transnational democracy.^13^ Going for the latter, more optimistic scenario, Kjaer advises rebuilding the European democratic ship “in a step-by-step manner while still at sea”.^14^ With COVID-19, we argue that we should not wait for calm seas to start rebuilding work; consolidation can and should be done also during the storm. This is even more important if placed in the context of current debates surrounding on the one hand the wider challenges to democracy posed by pandemic management nationally and transnationally, and on the other hand, the rule of law crisis faced by the EU.

Against this background, we take issue with the legitimacy of COVID-19 EU soft law instruments. We analyse the procedures through which these instruments were adopted, with the goal of determining who was involved in the process of emergency soft law-making. As such, our analysis draws on the input/throughput/output legitimacy structure proposed by Scharpf^15^ and revised by Schmidt.^16^ The output dimension, understood as policy efficiency and outcomes, is deliberately left for further research, given the relatively young age of the instruments studied and the lack of comprehensive data. We understand input legitimacy as participation through “majoritarian institutions of electoral representation”,^17^ and, in this context, we find that the absence of the European Parliament in the process of COVID-19 soft law-making is worrying. In relation to throughput legitimacy, we are mostly interested in the dimensions of “transparency, inclusiveness and openness of governance processes”.^18^ In that connection, we acknowledge that the accountability of policy makers is a crucial element of throughput legitimacy but a complete discussion of this aspect exceeds the ambit of our paper.^19^ Instead, we focus primarily on the participation of the national level of governance, scientific bodies and other stakeholders in the process of soft law making.

In order to determine the levels of participation, we compiled a dataset comprising all soft law instruments issued up to August 2020 for dealing with COVID-19 which are listed on the Eur-Lex web page. The procedures for issuing soft law are not mentioned in the Treaty, yet they are sometimes listed in the text of the instrument itself or in press releases. A textual analysis of our dataset showed, as expected, low levels of parliamentary participation and consultation with national levels of governance, scientific bodies or other stakeholders. Sometimes, information related to consultations appears in press releases accompanying certain soft law, yet it is presented in an unsystematic way, impairing transparency.

The article proceeds as follows. Section II places the debate on legitimacy of EU COVID-19 soft law measures against the background of more general transnational discussions regarding the democratic credentials of public interventions to counteract the effects of the pandemic. As such, it shows that the weak legitimacy of soft law creates a danger for democratic slippage of the EU itself in times of COVID-19. Section III presents the results of our textual analysis of COVID-19 EU soft law. Section IV provides some concluding thoughts and policy recommendations.

## II. EU Soft law as a danger to the legitimacy of COVID-19 interventions

There is no secret that the current health crisis is generating a massive shock for many of the world’s political systems. This article contributes to the emerging rich body of literature unveiling the risk of democratic slippage in terms of the pandemic, and shows that, unfortunately, the EU reaction is not free from criticism. This is primarily because of the weak legitimacy credentials of the legal instruments chosen to tackle the COVID-19 crisis, which are for the most part soft law.

### 1. COVID-19 and democracy

Amat et al reviewed the literature on the political effects of natural disasters and economic shocks and noted that these can affect both democracies and autocracies.^20^ Most recently, the COVID-19 crisis was connected with the turmoil in Belarus, where it had the potential to topple the Lukashenko regime,^21^ resonating with previous research showing that negative shocks can contribute to democratisation.^22^ Similarly, democracies might also suffer, with trust and cooperation affected negatively by restrictions imposed to control the virus, such as social distancing.^23^ At the very basic level, pandemics can impact the organisation of elections, and such activities have been already disrupted and continue to be disrupted worldwide by COVID-19.^24^

What it is not yet settled however, is how far the effects of this shock will extend, and especially what is the impact for democracy. The crisis raises important dilemmas “between globalism and nationalism, between public health and civil liberties and between political and technocratic governance’”.^25^ All these are reflected in public intervention against the pandemic in the EU Member States. The empirical evidence gathered by Amat et al from Spain shows worrying trends. As COVID-19 reduces public trust in politicians, individuals appear to prefer technocratic governance and authoritarian leadership. Similarly, people appear ready to give up civil liberties in order to protect public health.^26^ Conversely, relying on a survey auspiciously fielded right before and right after the start of the lockdown in Western Europe, Bol et al conclude that trust in government and satisfaction with democracy increased following the COVID-19 crisis.^27^

Similar divergent accounts were registered in relation to individuals’ perceptions *vis-à-vis* European intervention. A German study shows that the COVID-19 crisis is likely to influence European integration positively, with citizens who were concerned with contacting the virus supporting a more cohesive EU.^28^ The Spanish study by Amat et al revealed a strong preference for national responses to the COVID-19 crisis. Coordinated European action, on the other hand, seems to be preferred by the population for tackling other global problems, such as climate change or terrorism.^29^

As to the ways in which the EU will exit the COVID-19 crisis, Fabbrini imagines three potential routes: one in which solidarity will prevail and will cohesively transform EU polity; a return to the market; or a slippage into autocracy and nationalism.^30^ An optimistic account is presented by Riddervold et al in a book on EU crises.^31^ Out of three possible scenarios to exit a crisis such as COVID-19, namely an EU breakup, a more integrated EU, or an EU muddling through crises, data suggest that the latter is most likely. Based on previous responses to the financial crisis, the migration crisis, Brexit and populism, Riddervold et al did not find evidence of institutional breakdowns, with EU institutions showing resilience and potentially even gaining more influence. Thus, even though initially the EU was slow to mobilise, there are high chances that it will muddle through just fine, potentially finding new creative ways to go forward, yet not excluding negative consequences for democracy in the long run.^32^

This brief account shows just how hard it is to predict the consequences of the COVID-19 crisis for the future of the EU. Yet the democratic concern is a real one, and it is shared by general emergency literature as well, with authors warning, even before 2020, that exceptional circumstances such as this one might be misused in order to consolidate the power of the executive.^33^ Delegation to the executive has occurred extensively during the COVID-19 crisis at the national level, for example the Irish Health Act 2020,^34^ delegating extraordinary powers to the Minister of Health, or the UK Coronavirus Act 2020^35^ granting new powers to the government in the area of public health.^36^ Such delegation has not been without controversy. The UK has a legislative framework that could have been used instead, namely the Civil Contingencies Act 2004.^37^ Blick and Walker argued that with the Coronavirus Act 2020 the UK government has avoided key constitutional safeguards and stripped the UK Parliament of important powers.^38^ Part of the problem, as correctly identified by Greene, was that many parliaments could not really sit during the crisis, due to various rules of procedure.^39^ However, the same cannot be said of the EU Parliament, which adapted to the prevailing system of work-from-home, and created, from late March, platforms for video-conferencing for large multilingual meetings as well as for remote voting.^40^

At the EU level there was no similar delegation of powers to the executive, as in the UK or the Republic of Ireland. Yet as we will come to show, the vast majority of instruments issued to deal with the COVID-19 crisis were soft law instruments issued by the EU’s executive body, the Commission. The critiques expressed above of the potential of the crisis to increase the weight of technocratic over democratic governance therefore also apply in relation to the EU. Issued with generally little to no involvement from stakeholders or the European Parliament, such instruments follow procedures that tend to circumvent the more legitimate but costly ways to make decisions. It is to these critiques that the article now turns.

### 2. Weak legitimacy credentials of soft law: not the most democratic in times of crisis?

EU soft law has been under the cross-fire almost since its inception. As early as 1968, the European Parliament was warning about the dangers associated with the proliferation, by the Council, of acts not mentioned in the Treaty, notably the circumvention of decision-making formalities, regarding the consultation of the Parliament and the right of initiative of the Commission.^41^ EU institutions’ recourse to soft law was also perceived to encroach on Member State competences, with the French Conseil d’État condemning the profusion of Council decisions and resolutions, as well as of Commission communications.^42^ More recently, in its 2007 Resolution, the European Parliament took a very critical approach, holding that “the use of soft law is liable to circumvent the properly competent legislative bodies, may flout the principles of democracy and the rule of law under Article 6 of the EU Treaty, and also those of subsidiarity and proportionality”.^43^

Such objections have been explained by Dawson as “inter-institutional wrangling”, and rather harmless with regard to the legitimacy of European law-making.^44^ As argued by Snyder, the European Parliament’s involvement in the decision-making process could even increase through the intermediary of soft law measures such as inter-institutional agreements.^45^ Preparatory and informative soft law instruments might fulfil an important function in the pre-legislative stage, because it is through these means that the Parliament is informed and consulted on future legislation.^46^ Yet Eislet and Slominski disagree, and consider soft law detrimental to the European Parliament. They show that, regardless of the timid benefits described above, the bargaining power of the Parliament remains the same, as the outcome of the final negotiations on legislation can depart from the content of the inter-institutional agreement.^47^

With regard to stakeholder consultations in soft law decision-making, the relevant procedures are famously unsystematic, a problem that is neither new nor EU-specific.^48^ As noted by Baldwin, “consultative procedures are less rigorously adopted and structured in relation to rules whose binding nature is uncertain”.^49^ Writing about the open method of communication (OMC), Kröger noted that democratic credentials were shallow in practice given the informality of stakeholder participation, which resulted in an uneven representation that disproportionately favoured technocratic and bureaucratic views as opposed to substantive political or functional ones.^50^ A major downside identified in this regard was the untransparent character of the procedures and the lack of judicial accountability. Writing about soft law in the environmental field, Scott reached similar conclusions, noting that public consultations are generally happenstance, and that there is little information as to how they occur.^51^

Kajer notes that the increasing importance of informal methods of decision-making and of new governance is a crisis of legally constituted public power. In this process, the normative integrity and the functional capacity of the law is challenged. Speaking about the informal transfer of fiscal policy from Member States to the EU, Kjaer warns about a potential circumvention of democratic, law-based decision-making processes.^52^ This is because the Europeanisation of fiscal policy undermines the handling of fiscal policy through democratic decision-making ways at the national level, which is especially problematic given that such legitimate law-making cannot be replicated at the EU level. To a certain extent, the same reflections can also apply in relation to the current COVID-19 crisis management through (informal) soft law, and we argue that in this context the choice of legal instrument matters greatly.

## III. The legitimacy of COVID-19 soft law

The virtues of soft law in catalysing cooperation have been praised since the time of the A/H1N1 pandemic, as it leaves enough margin for states to construct their responses in accordance with national specificities.^53^ In the case of COVID-19, faced with an initial lack of cooperation from the Member States, the EU ultimately attempted to achieve convergence through the intermediary of an ever-increasing amount of soft law. The instruments setting the scene were, among others, a Communication on a coordinated economic response to the COVID-19 outbreak,^54^ dealing with the immediate response to the crisis, as well as a Joint Statement from the European Council^55^ followed up by two Roadmaps by the Council and the Commission^56^ on strategies and measures to end the lockdowns. These instruments, mainly of a steering nature, were soon followed by a substantive body of soft law, issued mostly by the European Commission under unprecedented time pressure.

These instruments cover various areas, each pertaining to different parcels of EU power. For instance, public health remains essentially a national competence, with a limited number of specific areas where the Union can intervene in order to support, coordinate or encourage policies and cooperation as per Article 168 TFEU. Researchers argue that the EU has more competences than one can discern at first sight in order to tackle the pandemic.^57^ However, Article 168(5) TFEU specifically provides for (formal) acts adopted under the ordinary legislative procedure – and not soft law – in order to improve human health and to combat major cross-border health scourges.

Furthermore, soft law was also issued in areas of exclusive EU competence – such as state aid or competition, where such instruments are a common feature of governance, and are relied on in order to interpret hard law. Yet the line between interpreting and adding to hard law provisions is famously thin,^58^ and the potential of such instruments to slip into the realm of ultra vires is high. Finally, it is tricky to regulate through soft law topics straddling between fundamental rights on the one hand and public health on the other. An example is contact tracing, which needs to be constructed in a proportional fashion both from a technical and regulatory perspective in order to strike the delicate balance between these two ideals.^59^ In this regard, authors have recommended that the EU and national levels work together towards suitable national implementation and flexible interpretation of central EU guidance.^60^

Seeking to address potential remedies to the legitimacy deficit of soft law, and trying to fill an important gap in the literature providing empirical evidence on the participatory reality of EU soft law-making,^61^ this article analyses the procedures through which COVID-19 EU soft instruments were issued. Section III.1 gives a brief account of our methods, with results presented in Section III.2 below.

### 1. A word on methodology

We worked on a dataset that we established in August 2020 by running a search through the 383 documents that the Eur-Lex database qualifies as “COVID-19 EU law”.^62^ Among these documents, we have selected the measures that, according to the definition provided in our Introduction, qualify as “soft law” measures. A total of 197 documents were identified. Table 1 provides an overview of the types of soft law measures that have been examined.

In our analysis, we agree that the legitimacy of soft law should be evaluated according to “dynamic” criteria, making use of national or subnational channels of governance, including peer review, and relying on hybrid combinations of traditional “Community” law and soft law.^63^ Van Dam suggested the publication of soft law instruments, and the creation of a democratic decision-making process for soft law, including greater national involvement in soft law-making.^64^ A similar conclusion was reached by Borras and Radaelli who used *demoi*-cratic standards to assess the OMC. These standards apply to transnational settings and purport the interaction between citizens and states in European law-making processes.^65^ Reviewing various studies, Borras and Radaelli reached the conclusion that the “*demoi*-cratic quality of the OMC hinges on the *demoi*-cratic quality of domestic institutions”.^66^ On the basis of this literature, we searched our dataset to identify traces of consultations between the Commission and various other bodies, namely:

1. The European Parliament
2. National authorities, including specialised administrations such as competition authorities, network industry regulators, etc
3. Relevant EU or national scientific bodies or agencies
4. Other stakeholders – such as NGOs, or businesses.

In particular, we have searched whether any of the keywords “stakeholder*”, “particip*”, “consult*” recurred in any of the identified soft law documents. We have further excluded documents in which these terms are used, but not in any of the four dimensions presented above. That reduced the sample to 27 soft law measures, divided as in Table 2.

Section III.2 outlines our findings. However, the limitations of our research need to be spelled out at the outset. Because of our reliance on keywords, the results cannot capture situations in which some sort of consultation or participation has occurred but the document itself either does not refer to it at all, or refers to it in different terms than those that we used for our search. Such is the situation of the Temporary Framework on State Aid.^67^ In this case, the Commission informed the general public about the consultations it carried out through the intermediary of press releases. Such unsystematic recording of the decision-making process is a finding in itself, perhaps raising issues related to institutional openness and transparency. For illustration purposes, the next subsection also looks at the consultations for the Framework, as they transpire from the press releases. In the interest of openness, we conclude that the Commission should make public, as far as possible, the text of these consultations, even if ex post factum.

### 2. Findings

The most significant finding – or absence – is that the European Parliament is, as expected, non-existent in the soft law-making processes. This might seem extraordinary given that, in a Resolution, the Parliament itself called for the intervention of various EU bodies to set up a coordinated action to combat the pandemic.^68^ Parliament appears attentive to the various topics dealt with COVID-19 soft law measures. For instance, the issue of data protection and COVID-19-related apps was one of the topics of the same parliamentary Resolution.^69^

From a sheer quantitative perspective, it can be observed that direct references to participation and consultation occur in a very small minority of COVID-19 EU soft law measures (27), amounting to less than 15% of the total. At a closer look, only six documents provide the information that prior consultation with EU or national authorities or with relevant stakeholders was carried out. Of those six, only two directly concern the management of the emergency situation created by COVID-19,^70^ with the remaining four only peripherally touching upon the pandemic.^71^ In the two soft law measures directly relating to the situation generated by COVID-19, reference is made to the fact that the European Center for Disease Prevention and Control was consulted prior to the adoption of the measures.

It thus emerges that the participatory and consultative dimension of COVID-19-related EU soft law is close to nil. More significantly, absent the European Parliament, the democratic input in the decision-making process has been non-existent. All is not entirely bleak, as in certain instruments, discussed below, the Commission promises to undertake consultations (i); other instruments set up national and European feedback loops similar to the open method of co-ordination (ii); and finally, traces of consultations can be found in press releases accompanying soft law instruments (iii).

#### a. Promises to undertake consultations

We found 22 measures in which the Commission pledges to consult either stakeholders or Member States at later stages of the implementation of the activities foreseen in the relevant soft law measure, or where the Commission encourages Member States to set up consultation procedures or ensure participatory processes. For example, in the Communication from the Commission titled “Guidelines on seasonal workers in the EU in the context of the COVID-19 outbreak”,^72^ the Commission invites Member States to consider, among other matters, the possibilities of including seasonal workers in consultation and participation mechanisms dealing with questions relating to safety and health at work. In the same document, the Commission pledges to continue working with EU-OSHA to collect information on good practices in occupational health and safety aspects affecting seasonal workers, and to make them available to the relevant stakeholders at national and EU levels, including through a dedicated information campaign targeted at these workers.

Similarly, in the Commission Communication titled “EU Guidance for the progressive resumption of tourism services and for health protocols in hospitality establishments – COVID-19”,^73^ the Commission pledges to continue working with Member States’ public authorities, tourism stakeholders and international organisations to facilitate the implementation of the guidance, and encourages Member States authorities to work closely with stakeholders in the elaboration of infection prevention and control measures and protocols.

#### b. Soft law providing for the cooperation with national levels of governance

A more systematic cooperation with national levels of governance is sometimes required by COVID-19 soft law. For example, the Commission recommendation on a common EU toolbox for the use of technology and data to address the COVID-19 crisis (the Commission Toolbox)^74^ replicates new governance mechanisms, such as the OMC. This instrument laid the foundations for the adoption, *together* with the Member States, of a toolbox to deal with the pandemic, aiming at coordinating approaches to using mobile applications for social distancing, warning, preventing and contact tracing, as well as using data to model and predict the evolution of the virus. The recommendation provided for mechanisms roughly similar to the European Semester or OMC cycles, whereby countries needed to report on the actions taken, with the Commission likely to issue further recommendations upon assessment.

As a result of these processes, several instruments were agreed, such as interoperability guidelines,^75^ technical specifications^76^ and the set-up of the gateway service.^77^ While future research will undoubtedly look into the ways in which these instruments were adopted, for the purposes of our article it is important to note that the Commission Toolbox catalysed the cooperation of the national level of governance, while bringing to the forefront networks of national authorities, such as eHealth.^78^

#### c. The example of the Temporary Framework on State Aid: consultations mentioned in the press releases

Sometimes, as happened in the case of the Temporary Framework on State Aid, one can see from various press releases that the Commission did undertake consultation, at least with Member States, probably at ministerial level. The state aid policy of the European Commission came very much into the spotlight during the COVID-19 crisis, as Member States needed to come to the rescue of their economies severely hit by the public health emergency and the various lockdowns. As provided for in Article 107(1) TFEU, state aid is in principle prohibited in the EU, and individual measures need to be approved by the European Commission. In deciding on exemptions, the Commission follows the regime of Article 107(2) and (3), enjoying the discretion to determine the compatibility of various projects with the internal market. In particular, Article 107(3)(b), excepting aid to remedy a serious disturbance in the economy of a Member State from the Treaty prohibition on state aid has been instrumental during the pandemic. The need for an EU-wide application of this exception was soon obvious, and the Commission issued a Temporary Framework^79^ providing for a quicker approval process. Indeed, as a follow up to the Framework, numerous measures have been approved by the Commission – and published on the website.^80^

A Statement from Commissioner Vestager^81^ informs us that work on the document was accelerated by the measures adopted by Member States. A draft was sent to the Member States for consultation only three days before the publication of the Framework itself. The full text of the consultation document is not publicly available, and it is impossible to gauge the level of national participation or participation of the institutions of the Member States involved. However, the press release contains a summary, from which it emerges that the initial threshold provided by the Commission for direct grants or tax advantages was lower than it was in the final text.^82^

The rapidly evolving situation prompted the need to update the Framework for the first time only two weeks later. Again, the full consultations are not published on the website of the Commission, but by comparing the accompanying Commission Statement^83^ with the final version of the text published only one week later we can see that the main points proposed by the Commission made it into the amended Framework.^84^

The second amendment concerns the extension of the Framework to recapitalisation measures, with a consultation document sent to the Member States only five days after the publication of the first amendment.^85^ The Commission was planning to have the updated Framework ready by the following week, but publication occurred one month later, understandably so given the complexity of issues at stake – and perhaps, also due to the extensive discussions with the Member States.^86^ There is still little transparency regarding the consultations undertaken, what voices were heard and how these voices were represented in the final text.

The third amendment followed some two months later.^87^ The Statement^88^ announcing consultations summarises the intention to further support micro, small and start-up companies and incentivise private investments, with the third amendment adopted along these lines. However, there is still little transparency as to how such consultations were conducted.^89^

Biondi argues that, given the urgency of this health crisis, the Commission should not be blamed for not having consulted widely.^90^ Similarly, Greene makes a powerful case that, with pandemics, exceptional emergency measures, even if put in place by the executive, are likely to have only a minimal impact on the powers of the democratically elected legislature. He argues that the restrictions of rights and liberties are bound to be more limited than in the case of terrorism emergency measures, which are usually taken against a targeted group, and tend to be in place in the long term. In case of pandemics, restrictions cover the entire population, which makes it difficult to sustain them in the long run, given the reduced popular support as well as their high costs. Thus, with COVID-19, one can expect a rush to normalcy before the danger is really over, rather than a disproportionate continuation of restrictive policies by the executive.^91^ We only partly agree with these positions. First, while comprehensive consultations of all stakeholders (such as businesses) are difficult to conduct in emergency circumstances, the experiences of drafting and amending the state aid Framework show that some form of exchange with the Member States at the ministerial level is always possible. We argue that in order for legitimacy to be preserved it is important for European institutions, and the Commission as main soft law-maker, to be as open as possible, for instance, the proceedings of the consultations that were undertaken need to be published as soon as feasible. Second, the complete lack of parliamentary involvement is problematic even if it is for a short period of time and concerns only temporary measures. It would be indeed difficult to determine a certain *de minimis* temporary rule for the infringement of democratic principles. Besides, it is not excluded that emergency measures could have important institutional spill-over effects after the crisis, entrenching the power of the executive or destabilising the institutional balance.

## IV. Conclusions and recommendations

The starting point for this article was that the democratic credentials of EU soft law are a well-known problem for this type of regulatory instrument, nationally and transnationally. Yet the use of soft law to deal with crisis situations such as COVID-19 presents even more acute dangers for democracy in the EU. Praised for its flexibility and fast adoption procedures, soft law scores typically rather low with respect to transparency and openness in its adoption procedures. The article empirically tested this position with respect to soft law issued in the context of the COVID-19 crisis. As shown above, the democratic credentials of EU COVID-19 soft law are extremely low, as it was issued with no involvement from the European Parliament and with minimal and unsystematic consultation with stakeholders, national authorities and scientific bodies.

This is problematic for the very efficacy of the emergency soft law. Given that soft law is not legally binding, top-down enforcement through courts or the European Commission is difficult.^92^ In such cases, the trust of the population needs to be enlisted in order to ensure voluntary compliance. One solution for trust is institutional openness, understood both as a requirement to transparently communicate to the public the various rules and processes,^93^ and as participation.^94^ Also, important safeguards for the rule of law, such as parliamentary and judicial controls, were deemed crucial for the effectiveness of emergency measures.^95^ Accountability, one of the dimensions of throughput legitimacy according to Schmidt, comes thus to the forefront. Writing about Norway, Christensen and Laegreid note that the key to crisis management lies in a dynamic combination of democratic legitimacy and government capacity.^96^ Citizen trust has been fuelled in Norway through a transparent collaboration between political, administrative and professional fora. Interestingly, non-binding guidance was employed for the stricter measures, and cooperation and consensus worked better than coercion.^97^ As observed by Christensen and Laegreid, communication with the public was paramount in the Norwegian case, suggesting that transparent approaches to tackling the pandemic are more successful.

What’s left for the EU to do now? Pontificating that the Parliament should have been involved in the issuing of soft law, or that stakeholders and the national levels of governance should have been consulted more thoroughly, is of little help if no solutions are offered for future crisis measures, potentially by adopting hard law outlining a detailed procedure to deal with emergencies. Obviously, measures to tackle pandemics need to be taken in a swift manner. Yet short deadlines could be devised for stakeholder consultations and parliamentary input could be sought quickly through the urgency procedure provided for in Article 163 of the Rules of Procedure (an article that has been used for several Regulations). Examples from the Member States show successful involvement from parliaments in tackling the crisis. The Finnish Parliament, for example, has been far from excluded from the management of the crisis even though day-to-day measures have been implemented mainly through recommendations.^98^

As for the measures that have already been taken, there is still time to mend the legitimacy of COVID-19 soft law and to increase public trust. First, in the short term, where the Commission did decide to consult stakeholders (such as private organisations and businesses) or national authorities (such as competition authorities, or other sector regulators), the relevant consultation documents, where available, should be published to increase the transparency of the process. Second, whenever the Commission pledged to consult the Member States, it should follow up on this promise and also communicate about its action and about the actors involved. Similarly, processes such as the consultations and reporting cycle put in place by the Commission Toolbox need to be monitored and, if successful, the lessons learnt from this process should be replicated. If the Union is to adopt legislation on how to deal with future emergencies, one of the guiding principles could be, for instance, that national levels of governance are involved in the decision-making processes. This can be done through formal consultations of the national parliaments, national administrations or regulatory authorities, but also by putting in place mechanisms that would foster rapid informal cooperation and mutual learning.

To conclude, while consultation and participation in soft law-making might not be the “silver bullet” that will rescue the EU from its democratic deficit, we argue that enhancing the transparency and openness of the process of adopting soft law measures is certainly a step in the right direction and might ultimately also increase acceptance of the measures thus enacted. This finding does not apply only to emergency measures, but, in the long run, more work is needed to increase transparency and participation in EU soft law.^99^

**Table 1.** Overview of COVID-19 EU soft law measures

Notices	68
Reports	32
Communications	30
Recommendations for a recommendation	29
Recommendations	14
Conclusions	7
Staff working documents	5
Guidelines	4
Joint communications	4
Opinions	2
Announcements	1
Statements	1

**Table 2.** Overview of COVID-19 EU soft law measures that foresee participation or consultation

Notices	10
Reports	1
Communications	10
Recommendations for a recommendation	0
Recommendations	1
Conclusions	1
Staff working documents	0
Guidelines	0
Joint communications	4
Opinions	0
Announcements	0
Statements	0

## Footnotes

1 F Snyder, “The Effectiveness of European Community Law: Institutions, Processes, Tools and Techniques” (1993) Modern Law Review 64.

2 G de Stefano, “Covid-19 and EU Competition Law: Bring the Informal Guidance On” (2020) Journal of European Competition Law & Practice 121–23.

3 Temporary Framework to support the economy in the context of the coronavirus outbreak OJ C 91I, 20 March 2020, 1–9.

4 Coronavirus: Commission Statement on consulting Member States on proposal to prolong and adjust State aid Temporary Framework <ec.europa.eu/commission/presscorner/detail/en/STATEMENT_20_1805>.

5 Communication from the Commission Guidelines on seasonal workers in the EU in the context of the COVID-19 outbreak 2020/C 235 I/01.

6 Communication from the Commission Guidelines on the progressive restoration of transport services and connectivity – COVID-19 2020/C 169/02.

7 Communication from the Commission EU Guidance for the progressive resumption of tourism services and for health protocols in hospitality establishments – COVID-19 2020/C 169/01.

8 Proposal for a Council Recommendation on a coordinated approach to the restriction of free movement in response to the COVID-19 pandemic COM(2020) 499 final.

9 O Ştefan, “COVID-19 Soft Law: Voluminous, Effective, Legitimate? A Research Agenda” (2020) European Papers Forum <www.europeanpapers.eu/en/europeanforum/covid-19-soft-law-voluminous-effective-legitimate>; O Ştefan, “The Future of European Union Soft Law: A Research and Policy Agenda for the Aftermath Of COVID-19” (2020) 7(2) Journal of International and Comparative Law.

10 C Kilpatrick, “On the Rule of Law and Economic Emergency: The Degradation of Basic Legal Values in Europe’s Bailouts” (2015) 35(2) Oxford Journal of Legal Studies 325–53.

11 M Dawson and F de Witte, “Constitutional Balance in the EU” (2013) 76(5) Modern Law Review 817–44.

12 P Kjaer, “European crises of legally-constituted public power: From the ‘law of corporatism’ to the ‘law of governance’” (2017) 23(5) European Law Journal 417–30.

13 J Habermas, “The Crisis of the European Union in the Light of a Constitutionalization of International Law” (2012) 23(2) European Journal of International Law 335–48.

14 Kjaer, supra, note 12, 417–30.

15 F Scharpf, *Governing in Europe: Effective and Democratic* (Oxford University Press 1999).

16 V Schmidt, “Democracy and Legitimacy in the EU Revisited: Input, Output and ‘Throughput’” (2013) 61(1) Political Studies 2.

17 As summarised by Schmidt, ibid, 5.

18 V Schmidt and M Wood, “Conceptualizing Throughput Legitimacy: Procedural Mechanisms of Accountability, Transparency, Inclusiveness and Openness in EU Governance” (2019) 97(4) Public Administration 727–40, 728.

19 For instance, judicial accountability. For thoughts on the other dimensions of the Scharpf/Schmidt taxonomy, see Ştefan, supra, note 9.

20 F Amat et al, “Pandemics meet democracy. Experimental evidence from the COVID-19 crisis in Spain” (2020) SocArXiv <DOI:10.31235/osf.io/dkusw> 4 et seq.

21 A Hansen, “Slow Stagnation, Sudden Revolution? Post-Covid-19 Prospects for Political Change in Russia and Belarus”, Norwegian Institute for International Affairs (NUPI) (2020) <www.jstor.org/stable/resrep25735>.

22 D Acemoglu and JA Robinson, “A theory of political transitions” (2001) American Economic Review 938–63; TS Aidt and G Leon, “The democratic window of opportunity: Evidence from riots in Sub-Saharan Africa” (2016) 60(4) Journal of Conflict Resolution 694–717; M Brückner and A Ciccone, “Rain and the democratic window of opportunity” (2011) 79(3) Econometrica 923–47.

23 A Aassvie et al, “Epidemics and Trust: The Case of the Spanish Flu” (2020) Working Papers 661 IGIER (Innocenzo Gasparini Institute for Economic Research), Bocconi University.

24 T Landman and L di Gennaro Splendore, “Pandemic democracy: elections and COVID-19” (2020) 23(7–8) Journal of Risk Research <DOI:10.1080/13669877.2020.1765003>.

25 Amat et al, supra, note 20, 7.

26 ibid, 22.

27 D Bol et al, “The effect of COVID-19 lockdowns on political support: Some good news for democracy?” (2020) European Journal of Political Research <DOI:10.1111/1475-6765.12401>.

28 JN Krehbiel, and S Cheruvu, “How Do Crises Affect Public Support for European Integration? The COVID-19 Pandemic and German Public Opinion”, conditionally accepted for Journal of Political Institutions and Political Economy <static1.squarespace.com/static/5d129089df7c230001b9dd2f/t/5f2b41245e640d7a47558c1c/1596670244824/Cheruvu_Krehbiel_3.pdf>.

29 Amat et al, supra, note 20, 25.

30 F Fabbrini, “The Future of the EU after Brexit, and COVID-19” (2020). BRIDGE Network – Working Paper 3, available at SSRN: <ssrn.com/abstract=3604111 or <dx.doi.org/10.2139/ssrn.3604111>.

31 M Riddervold et al (eds), *The Palgrave Handbook of EU Crises* (Palgrave 2021, forthcoming).

32 J Trondal et al, “What previous crises tell us about the likely impact of Covid-19 on the EU”, LSE European Politics and Policy, 20 April 2020, <blogs.lse.ac.uk/europpblog/2020/04/21/what-previous-crises-tell-us-about-the-likely-impact-of-covid-19-on-the-eu/>.

33 C Bjørnskov and S Voigt, “The Architecture of Emergency Constitutions” (2018) 16(1) International Journal of Constitutional Law 101, 125.

34 Health (Preservation and Protection and other Emergency Measures in the Public Interest) Act 2020 <www.irishstatutebook.ie/eli/2020/act/1/enacted/en/html>.

35 Coronavirus Act 2020 <www.legislation.gov.uk/ukpga/2020/7/contents/enacted>.

36 A Greene, Emergency *Powers in a Time of a Pandemic* (Bristol University Press 2020) p 55.

37 Civil Contingencies Act 2004 <www.legislation.gov.uk/ukpga/2004/36/contents>.

38 A Blick and C Walker, “Why did government not use the Civil Contingencies Act?” The Law Society Gazette (2 April 2020) <www.lawgazette.co.uk/legal-updates/why-did-government-not-use-the-civil-contingencies-act/5103742.article>.

39 Greene, supra, note 36, 57 et seq.

40 T Feeley, “European Parliament and Covid-19”, UK in a Changing Europe Commentary (20 April 2020) <ukandeu.ac.uk/european-parliament-and-covid-19/>.

41 Résolution du Parlement européen du 8 mai 1969, sur les actes de la collectivité des États membres de la Communauté ainsi que les actes du Conseil non prévus par les traités adoptée à la suite du rapport fait au nom de la Commission juridique par M. Burger [1969] OJ C63/18.

42 Conseil d’État, Rapport Public 1992, collection “Études et documents”, Documentation française 44, 1993, Paris, 22–23 cited in S Leclerc, “Les communications de la Commission et le marché intérieur. A propos de l’arrêt rendu par la Cour de justice des Communautés européennes le 20 mars 1997 dans l’affaire C-57-95, Rec (1997), p I-1640 à I-1652” (1998) Cahiers de droit européen 161, 163.

43 Resolution of the European Parliament “on the institutional and legal implications of the use of soft law instruments”, 2007/2028(INI), at 1.

44 M Dawson, “Soft Law and the Rule of Law in the European Union: Revision or Redundancy?” (2009) EUI Working Papers (RSCAS) 2009/24 <ssrn.com/abstract=1415003>, 5.

45 F Snyder, “Interinstitutional Agreements: Forms and Constitutional Limitations”, in G Winter (ed), *Sources and Categories of European Union Law* (Nomos Verlag 1996) p 459.

46 L Senden, *Soft Law in European Community Law* (Hart Publishing 2004) p 483.

47 See the discussion in I Eiselt and P Slominski, “Sub-Constitutional Engineering: Negotiation, Content, and Legal Value of Interinstitutional Agreements in the EU” (2006) 12(2) European Law Journal 209.

48 See in general on this point S Smismans, “New modes of governance and the participatory myth” in R Bellamy and A Palumbo (eds), *From Government to Governance. The Library of Contemporary Essays in Political Theory and Public Policy* (Ashgate 2010) pp 271–92.

49 R Baldwin, *Rules and Government* (Clarendon Press 1995) p 284.

50 S Kröger, “The End of Democracy as We Know it? The Legitimacy Deficits of Bureaucratic Social Policy Governance” (2007) 29(5) Journal of European Integration 565–82.

51 J Scott, “In Legal Limbo: Post-legislative Guidance as a Challenge for European Administrative Law” (2011) 48(2) Common Market Law Review 329, 336.

52 PF Kjaer, “European crises of legally-constituted public power: From the ‘law of corporatism’ to the ‘law of governance’” (2017) 23(5) European Law Journal 417–30.

53 B Bennett and T Carney, “Law, ethics and pandemic preparedness: the importance of cross-jurisdictional and cross-cultural perspectives” (2010) 34(2) Australian and New Zealand Journal of Public Health 106.

54 Commission Communication of 13 March 2020 on a coordinated economic response to the COVID-19 Outbreak COM(2020) 112 final.

55 Joint statement of the Members of the European Council, 26 March 2020.

56 The Joint European Roadmap towards lifting COVID-19 containment measures <ec.europa.eu/info/sites/info/files/communication_-_a_european_roadmap_to_lifting_coronavirus_containment_measures_0.pdf>; Roadmap for Recovery <www.consilium.europa.eu/media/43384/roadmap-for-recovery-final-21-04-2020.pdf>.

57 K Purnhagen et al, “More Competences than You Knew? The Web of Health Competence for EU Action in Response to the COVID-19 Outbreak” (2020) 11(2) EJRR 297.

58 Scott, supra, note 51, 329.

59 C Cattuto and A Spina, “The Institutionalization of Digital Public Health: Lessons Learned from the COVID-19 App” (2020) 11(2) EJRR 228.

60 Greene, supra, note 36.

61 See however, with respect to EU agencies, S Vaughan, “Differentiation and Dysfunction: An Exploration of Post-Legislative Guidance Practices in 14 EU Agencies” in *Cambridge Yearbook of European Legal Studies* 66-91 (CUP 2015); E Korkea-aho, “Laws in Progress? Reconceptualizing Accountability Strategies in the Era of Framework Norms” (2013) 2(02) Transnational Environmental Law 363–85; P Rocca and M Eliantonio, “European Union Soft Law by Agencies: an Analysis of the Legitimacy of their Procedural Frameworks” in M Conticelli et al (eds), *EU Executive Governance: Agencies and Procedures* (Giappichelli 2019) pp 177–203.

62 See <eur-lex.europa.eu/search.html?orDNGroup=CM%3DCOVID19&qid=1455633998284&CASE_LAW_SUMMARY=false&DTS_DOM=ALL&typeOfActStatus=OTHER&type=advanced&lang=en&SUBDOM_INIT=ALL_ALL&DTS_SUBDOM=ALL_ALL>.

63 M Dawson, “Soft Law and the Rule of Law in the European Union: Revision or Redundancy?” (2009) EUI Working Papers (RSCAS) 2009/24 <ssrn.com/abstract=1415003> 15–16.

64 C van Dam, “De Doorwerking van Europese Administratieve Soft Law: In Strijd Met Nederlandse Legaliteit?” (2013) Netherlands Administrative Law Library <www.nall.nl/tijdschrift/nall/2013/01/NALL-D-12-00008>.

65 F Cheneval et al, “Demoicracy in the European Union: principles, institutions, policies” (2015) 22(1) Journal of European Public Policy 1–18.

66 S Borras and CM Radaelli, “Open Method of Co-ordination for Demoi-cracy? Standards and Purposes” (2015) Journal of European Public Policy 129, 141.

67 Communication from the Commission Temporary Framework for State Aid Measures to Support the Economy in the Current COVID-19 Outbreak 2020/C 91 I/01, C/2020/1863 [2020] OJ C 91I/1–9.

68 European Parliament resolution of 17 April 2020 on EU coordinated action to combat the COVID-19 pandemic and its consequences, P9_TA(2020)0054.

69 ibid.

70 Communication from the Commission towards a phased and coordinated approach for restoring freedom of movement and lifting internal border controls – COVID-19, C(2020) 3250 final; Joint European Roadmap towards lifting COVID-19 containment measures, 2020/C 126/01.

71 Communication from the Commission amending the Annex to the Communication from the Commission to the Member States on the application of Articles 107 and 108 of the Treaty on the Functioning of the European Union to short-term export-credit insurance 2020/C 101 I/01; Communication from the Commission to the European Parliament, the Council, the European Economic and Social Committee and the Committee of the Regions – EU strategy for a more effective fight against child sexual abuse COM/2020/607 final; Communication from the Commission to the European Parliament and the Council – State of play as regards the situation of non-reciprocity in the area of visa policy; Joint Communication to the European Parliament, the European Council, the Council, the European Economic and Social Committee and the Committee of the Regions – Eastern Partnership policy beyond 2020 Reinforcing Resilience – an Eastern Partnership that delivers for all COM/2020/119 final.

72 See supra, note 5.

73 See supra, note 6.

74 Commission Recommendation (EU) 2020/518 of 8 April 2020 on a common Union toolbox for the use of technology and data to combat and exit from the COVID-19 crisis, in particular concerning mobile applications and the use of anonymised mobility data C/2020/3300 (OJ L 114, 14 April 2020, 7–15).

75 eHealth Network, Interoperability guidelines for approved contact tracing mobile applications in the EU <ec.europa.eu/health/sites/health/files/ehealth/docs/contacttracing_mobileapps_guidelines_en.pdf>.

76 See the documents available at <ec.europa.eu/health/ehealth/key_documents_en#anchor0>.

77 Commission Implementing Decision (EU) 2020/1023 of 15 July 2020 amending Implementing Decision (EU) 2019/1765 as regards the cross-border exchange of data between national contact tracing and warning mobile applications with regard to combatting the COVID-19 pandemic C/2020/4934 OJ L 227I, 16 July 2020, 1–9.

78 For an outline of the increasing importance of this network in recent years see C Bérut, “The European Union as an opportunity: structures and uses of European soft law in French, Austrian and Irish eHealth policies” (2020) West European Politics <DOI:10.1080/01402382.2020.1739881>.

79 Communication from the Commission of 20 March 2020, Temporary Framework for State aid measures to support the economy in the current COVID-19 outbreak 2020/C 91 I/01.

80 See A Biondi, “Governing the Interregnum: State Aid rules and the COVID -19 Crisis” (2020) IV(2) Market and Competition Law Review.

81 Statement by Executive Vice-President Margrethe Vestager on a draft proposal for a State aid Temporary Framework to support the economy in the context of the COVID-19 outbreak, 17 March 2020 <ec.europa.eu/commission/presscorner/detail/en/statement_20_479>.

82 Communication from the Commission Temporary Framework for State aid measures to support the economy in the current COVID-19 outbreak 2020/C 91 I/01 C/2020/1863 OJ C 91I, 20 March 2020, 1–9.

83 Coronavirus: Commission Statement on consulting Member States on the proposal to extend State aid Temporary Framework 27 March 2020 <ec.europa.eu/commission/presscorner/detail/en/statement_20_551>.

84 Communication from the Commission – Amendment to the Temporary Framework for State aid measures to support the economy in the current COVID-19 outbreak 2020/C 112 I/01 C/2020/2215 OJ C 112I, 4 April 2020, 1–9.

85 Coronavirus: Commission Statement on consulting Member States on proposal to further expand State aid Temporary Framework to recapitalisation measures, 9 April 2020 <ec.europa.eu/commission/presscorner/detail/en/STATEMENT_20_610>.

86 Communication from the Commission – Amendment to the Temporary Framework for State aid measures to support the economy in the current COVID-19 outbreak C(2020) 3156 final <ec.europa.eu/competition/state_aid/what_is_new/sa_covid19_2nd_amendment_temporary_framework_en.pdf>.

87 Communication from the Commission Third amendment to the Temporary Framework for State aid measures to support the economy in the current COVID-19 outbreak 2020/C 218/03 C/2020/4509 OJ C 218, 2 July 2020, 3–8.

88 See <ec.europa.eu/commission/presscorner/detail/en/STATEMENT_20_1054>.

89 Press release 29 June 2020 State aid: Commission expands Temporary Framework to further support micro, small and start-up companies and incentivise private investments <ec.europa.eu/commission/presscorner/detail/en/ip_20_1221>.

90 Biondi, supra, note 80.

91 Greene, supra, note 36, p 66.

92 O Ştefan, “Soft Law and the Enforcement of EU Law”, in A Jakab and D Kochenov (eds), *The Enforcement of EU Law and Values: Ensuring Member States’ Compliance* (Oxford University Press 2017) pp 200–17.

93 K Lenaerts, “‘In the Union We Trust’: Trust – Enhancing Principles of Community Law” (2004) 41(2) Common Market Law Review 317–43.

94 D Curtin and J Mendes, “Transparence et Participation: des Principes Démocratiques pour l’Administration de l’Union Européenne” (2011) 1(137–8) Revue Française d’Administration Publique 101–21; A Meijer et al, “Open government: connecting vision and voice” (2012) 78(1) International Review of Administrative Sciences 10–29.

95 J Petrov, “The COVID-19 emergency in the age of executive aggrandizement: what role for legislative and judicial checks?” (2020) 8(1–2) Theory and Practice of Legislation <DOI:10.1080/20508840.2020.1788232>.

96 T Christensen and P Lægreid, “Balancing Governance Capacity and Legitimacy: How the Norwegian Government Handled the COVID-19 Crisis as a High Performer” (2020) 80(5) Public Administration Review <DOI:10.1111/puar.13241>.

97 ibid, 774–79.

98 M Scheinin, “The COVID-19 Emergency in Finland: Best Practice and Problem” (2020) <verfassungsblog.de/the-covid-19-emergency-in-finland-best-practice-and-problems/>. See also H Wenander, “Sweden: Non-binding Rules against the Pandemic – Formalism, Pragmatism and Some Legal Realism” (2021) European Journal of Risk Regulation <DOI:10.1017/err.2021.2>.

99 See also the policy recommendations of the European Network on Soft Law Research: M Eliantonio et al, *EU Soft Law in the Member States* (Hart Publishing 2021, forthcoming).

